# Research on the Multiscale Characterization and Performance of Basalt Fiber Powder-Modified Sasobit Warm-Mix Asphalt

**DOI:** 10.3390/ma19091708

**Published:** 2026-04-23

**Authors:** Yuhan Li, Zhaoyang Chen, Junwei Bi, Meisheng Shi

**Affiliations:** 1 School of Civil Engineering, Shandong University, Jinan 250002, China; 2 Shandong Key Laboratory of Technologies and Systems for Intelligent Construction Equipment, Shandong Jiaotong University, Jinan 250357, China; 3 School of Qilu Transportation, Shandong University, Jinan 250002, China

**Keywords:** basalt fiber powder, warm-mix asphalt, interfacial adhesion, rheological properties, pavement performance

## Abstract

Warm-mix asphalt (WMA) technology and basalt fiber modification have been increasingly applied in road engineering. However, conventional basalt fibers often disperse unevenly and tend to agglomerate. In this study, basalt fiber powder (BFP) was incorporated into a Sasobit-based WMA system and systematically compared with matrix asphalt, Sasobit-modified WMA, conventional basalt fiber-modified WMA, and styrene butadiene styrene (SBS)-modified asphalt. Multiscale characterization—including dynamic shear rheometry (DSR), bending beam rheometry (BBR), scanning electron microscopy (SEM), and nanoindentation—was conducted to elucidate rheological behavior and interfacial micromechanical responses. The corresponding Asphalt Concrete-13 (AC-13) mixtures were further evaluated through rutting tests, low-temperature bending tests, and moisture susceptibility tests. Results demonstrate that micronized BFP achieves more homogeneous dispersion within the asphalt matrix and may promote a more effective reinforcing morphology, significantly enhancing high-temperature deformation resistance while partially mitigating the low-temperature stiffness increase induced by Sasobit. Compared with conventional basalt fiber systems, BFP shows better stress relaxation capacity and interfacial mechanical response under the tested conditions. At the mixture level, the BFP–Sasobit system showed the best overall performance, with the dynamic stability increasing by 242.2% relative to the base asphalt mixture and the residual Marshall stability reaching 92.3%, while the low-temperature flexural strain increased by 33.3%. Overall, the findings suggest that morphology-controlled micronization provides a morphology-guided enhancement strategy for Sasobit-based warm-mix asphalt by promoting coordinated improvements across the rheological, micromechanical, and mixture scales.

## 1. Introduction

Warm-mix asphalt (WMA) technologies enable reduced mixing and paving temperatures, offering the dual benefits of lower energy consumption and emissions alongside improved workability. As such, WMA has emerged as a key low-carbon strategy in road construction [1,2]. Sasobit, a representative organic wax-based WMA additive, enhances coating and compaction by reducing binder viscosity at construction temperatures [3,4,5]. However, its structuring effect can increase binder stiffness and alter temperature susceptibility, potentially compromising cracking resistance and long-term durability under varying climatic and traffic conditions [6,7,8]. Consequently, a critical balance must be struck between constructability and in-service performance through targeted modification, ensuring that the inherent advantages of warm-mix technology are fully retained.

To address these challenges, fiber reinforcement has been widely explored as an effective strategy to enhance bridging effects, asphalt adsorption, and energy dissipation within the binder, thereby compensating for potential interfacial weakening associated with warm-mix technologies and improving the overall performance of WMA mixtures [9,10,11]. Among various fiber types, basalt fiber has attracted considerable attention due to its physicochemical compatibility with mineral aggregates, strong adhesion to asphalt, and growing acceptance as a sustainable alternative to polyester and lignin fibers [12,13,14,15]. Extensive laboratory investigations have been conducted to optimize fiber-related parameters, including content, length, and diameter. For instance, Tanzadeh et al. [16] demonstrated that basalt fibers contribute to binder stabilization and reduce draindown in porous asphalt; however, the effectiveness of such reinforcement is highly contingent upon uniform fiber dispersion. Using nano-CT and finite element analysis, Cheng et al. [17] further revealed that chopped basalt fibers (CBFs) tend to form bundles or agglomerates within the asphalt matrix, resulting in uneven spatial distribution and compromised reinforcement stability. Similarly, Hui et al. [12] noted that the relatively smooth surface of CBFs limits interfacial bonding with asphalt, potentially leading to slippage and diminished reinforcing efficiency in practical applications.

To improve interfacial adhesion and dispersion, chemical surface modification—such as coating basalt fibers with coupling agents—has been attempted, aiming to enhance chemical bonding and mechanical interlocking with asphalt [18,19,20]. Nevertheless, these approaches increase process complexity and cost, constraining their viability for large-scale implementation [21]. In response, recent research has shifted focus from merely optimizing fiber dosage and geometry toward tailoring fiber morphology. In particular, flocculent basalt fibers (FBFs) have been introduced to form a more open three-dimensional network, thereby increasing the effective contact area and promoting asphalt adsorption. This morphological feature strengthens interfacial interaction and contributes to improved deformation recovery and cracking resistance [22,23,24]. Moreover, evidence from warm-mix recycled asphalt systems suggests that morphology-dependent reinforcement can effectively delay crack initiation and propagation [25,26].

Despite extensive studies on warm-mix asphalt additives and fiber reinforcement, limited attention has been paid to whether modifier morphology, particularly micronized basalt fiber powder, can improve dispersion and interfacial interaction in Sasobit-based systems and thereby generate coordinated responses across binder and mixture scales. This constitutes the specific gap addressed in the present work.

To overcome these challenges, this study proposes a micronization strategy in which conventional basalt fibers are mechanically ground into basalt fiber powder (BFP) via controlled ball milling. The resulting BFP was incorporated into a Sasobit-based warm-mix asphalt (WMA) system to investigate its morphology-dependent reinforcement mechanisms. For comparative purposes, five binder systems were prepared: matrix asphalt, SBS-modified asphalt, Sasobit-modified WMA, conventional basalt fiber-modified WMA, and BFP-modified WMA. The rheological properties of these binders were systematically evaluated using dynamic shear rheometer (DSR) and bending beam rheometer (BBR) tests to assess high-temperature rutting resistance and low-temperature cracking performance. Scanning electron microscopy (SEM) and nanoindentation were further employed to characterize fiber dispersion morphology and interfacial micromechanical behavior. To bridge binder-level mechanisms with mixture-scale performance, corresponding AC-13 WMA mixtures were fabricated and subjected to rutting tests at 60 °C, low-temperature bending tests at −10 °C, and water immersion Marshall tests. This multiscale experimental framework enables a comprehensive understanding of how morphological modification of basalt fibers influences the performance of Sasobit-based WMA systems across different scales.

This study aims to examine whether the performance enhancement associated with morphology-controlled micronization can be interpreted mainly in terms of improved interfacial stress transfer, rather than a simple volumetric stiffening effect similar to that of conventional mineral fillers. In parallel, the potential synergistic interaction between BFP and Sasobit in warm-mix systems is systematically examined. The findings are expected to establish a feasible strategy for engineering durable and low-carbon asphalt pavements.

## 2. Materials and Methods

### 2.1. Materials

#### 2.1.1. Base Asphalt

The base asphalt selected for this study was a 70# Grade A road petroleum asphalt, which was used as the control and as the base for all modified asphalt binders. Sample preparation and testing were carried out strictly following the Standard Test Methods of Bitumen and Bituminous Mixtures for Highway Engineering [27]. The key properties of the base asphalt are listed in Table 1.

#### 2.1.2. SBS-Modified Asphalt

The physical and rheological properties of the SBS-modified asphalt applied in this study are detailed in Table 2.

#### 2.1.3. Warm Mix Agent

The warm-mix additive employed in this study was Sasobit. By lowering the viscosity of the asphalt binder, Sasobit improves flowability at reduced temperatures, thereby enabling lower construction temperatures. Its primary technical specifications are listed in Table 3.

#### 2.1.4. Basalt Fiber

The conventional performance properties of the basalt fiber (BF) are listed in Table 4.

#### 2.1.5. Aggregate

The coarse and fine aggregates used in the asphalt mixtures were basalt and limestone, respectively, and the mineral filler was prepared from finely ground limestone. The asphalt mixtures were designed with AC-13 gradation, corresponding to a nominal maximum aggregate size of 13.2 mm. The performance properties of all aggregates and filler conformed to the requirements of the Standard Test Methods of Bitumen and Bituminous Mixtures for Highway Engineering (JTG E20–2011).

### 2.2. Experimental Design

#### 2.2.1. Preparation of Basalt Fiber Powder (BFP)

Continuous basalt fibers (diameter: 9.0 μm) were dried at 105 °C for 4 h and then cut into 1.0 mm~2.0 mm segments. The resulting short fibers were ground in a planetary ball mill using zirconia balls (6.0 mm diameter) as the grinding medium. The ball-to-material mass ratio was 8:1, the rotational speed was 300.0 rpm, and the milling time was 2.5 h. Intermittent liquid nitrogen cooling was applied to maintain the grinding chamber temperature below 50 °C throughout the process. The milled product exhibited an observed characteristic size on the order of 8 ± 2 μm and consisted primarily of short fiber fragments with a reduced aspect ratio, rather than equiaxed mineral particles. A full quantitative characterization of particle size distribution and shape was beyond the scope of the present study and should be addressed in future work.

#### 2.2.2. Preparation of Modified Asphalt Samples

Sasobit warm-mix asphalt (SWMA) was first prepared by adding 3% Sasobit (by weight of the base asphalt) to 500.0 g of base asphalt heated to 160 °C, following established mix designs [28,29]. The mixture was sheared at 3000.0 rpm for 20.0 min at 130 ± 5 °C [28] and then conditioned in an oven at 130 °C for 0.5 h.

**Figure 1:**
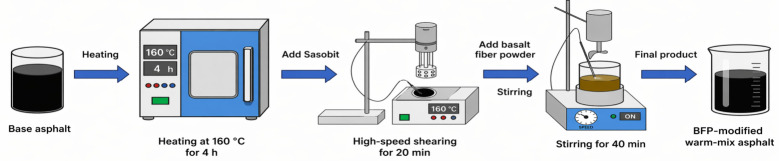
Schematic illustration of the preparation process for BFP-modified warm-mix asphalt.

Two fiber-modified binders were subsequently prepared using SWMA as the base. For BF-WMA, basalt fibers (BFs) were gradually incorporated into SWMA in three equal portions at a total dosage of 1% (by weight of binder). For BFP-WMA, basalt fiber powder (BFP) was added following the same three-portion procedure at an equivalent dosage of 1% (by weight of binder) in a separate batch [30]. The same dosage was intentionally used for BF and BFP in order to enable a direct comparison of morphology-related effects under a controlled dosage condition. Therefore, the present study focuses on morphology comparison rather than dosage optimization. The selected dosages were based on representative values reported in previous studies, and the same 1 wt.% dosage was used for BF and BFP to enable a direct comparison of morphology-related effects rather than dosage-related differences. After each addition, the blend was sheared at 1000 rpm at 140 °C for 40 min, followed by oven conditioning at 130 °C for 1 h to ensure stabilization and homogeneity. The preparation process is illustrated schematically in Figure 1.

#### 2.2.3. Dynamic Shear Rheometer (DSR) Tests for High-Temperature Performance

**Figure 2:**
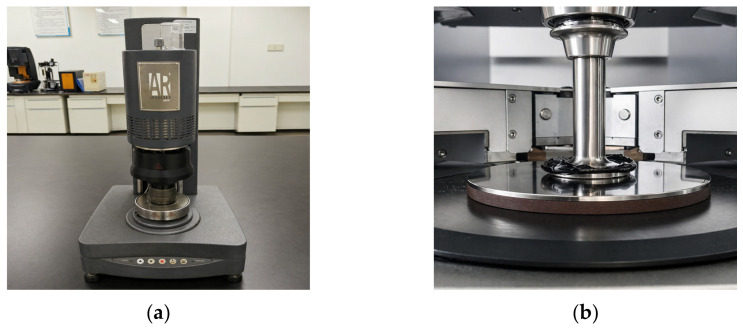
DSR test. (**a**) Dynamic shear rheometer. (**b**) Sample placement (before sample preparation).

Temperature sweep tests were performed using a dynamic shear rheometer (DSR, AR 2000ex, TA Instruments, NEW Castle, DE, USA) to characterize the rheological properties of the five asphalt binders: base asphalt, SBS-modified asphalt, Sasobit warm-mix asphalt (SWMA), basalt fiber-modified WMA (BF-WMA), and basalt fiber powder-modified WMA (BFP-WMA). The tests were conducted over a temperature range of 52 °C~82 °C at 6 °C intervals, with a constant angular frequency of 10 rad/s. The DSR testing protocol is illustrated schematically in Figure 2.

#### 2.2.4. Bending Beam Rheometer (BBR) Tests for Low-Temperature Performance

**Figure 3:**
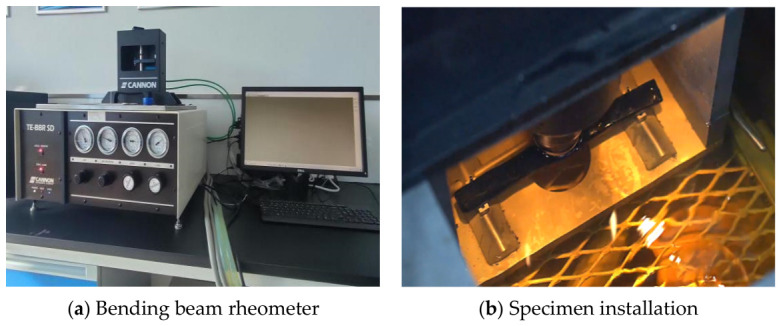
BBR test process.

Low-temperature rheological properties related to thermal cracking were characterized using a bending beam rheometer (BBR, TE-BB SD, CANNON Instrument Company, State College, PA, USA). Tests were conducted on the following five asphalt binders: base asphalt, Sasobit warm-mix asphalt (SWMA), basalt fiber-modified WMA (BF-WMA), basalt fiber powder-modified WMA (BFP-WMA), and SBS-modified asphalt. Measurements were performed at −12 °C, −18 °C, and −24 °C (6 °C intervals). Beam specimens (125.0 mm × 12.5 mm × 6.25 mm) were conditioned at the target temperature for 16 h prior to testing. The creep stiffness (*S*) and *m*-value were determined to evaluate low-temperature cracking resistance. The BBR test setup is illustrated schematically in Figure 3.

#### 2.2.5. Scanning Electron Microscopy (SEM) for Dispersion Morphology Characterization

**Figure 4:**
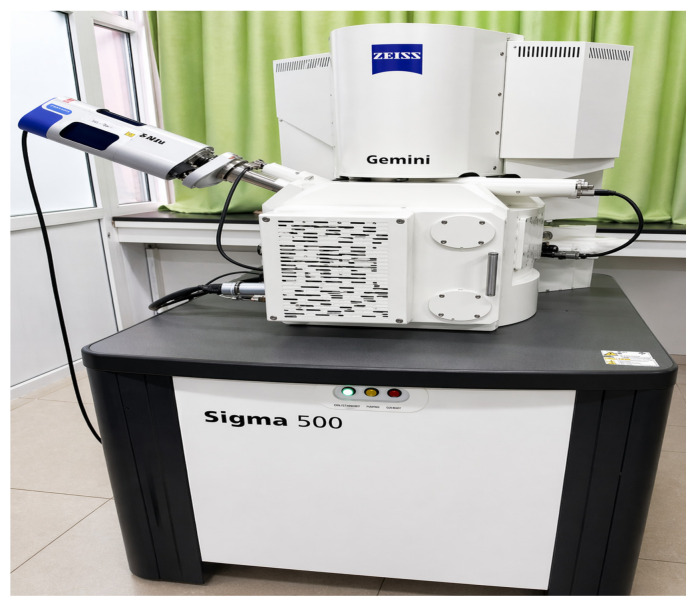
Scanning electron microscope.

The dispersion morphology of basalt fibers and basalt fiber powder within the warm-mix asphalt binder was characterized using a ZEISS Sigma 500 field emission scanning electron microscope (FE-SEM, Sigma 500, Carl Zeiss Microscopy GmbH, Oberkochen, Germany). Observations were conducted at an accelerating voltage of 3 kV~5 kV and a working distance of 6 mm~8 mm to ensure image clarity and accuracy. The SEM used in this study is shown in Figure 4.

#### 2.2.6. Nanoindentation for Micromechanical Property Characterization

The micromechanical properties of the asphalt binders were characterized using a nanoindentation system. Elastic modulus, a key parameter reflecting resistance to localized deformation, was measured for the following five binder types: base asphalt, SBS-modified asphalt, Sasobit warm-mix asphalt (SWMA), basalt fiber-modified WMA (BF-WMA), and basalt fiber powder-modified WMA (BFP-WMA).

Specimens for nanoindentation were prepared by cutting each asphalt binder sample into sections of approximately 1 cm × 1 cm. These sections were then mounted on the nanoindenter sample stage, and the surface was carefully leveled to ensure alignment with the reference columns at the four corners of the stage, thereby minimizing topographic effects on the measurements.

Following sample mounting, the stage was secured in the nanoindentation tester’s mounting bracket. The InView software (KLA Corporation, Milpitas, CA, USA; available online: https://www.kla.com/products/instruments/nanoindenters?utm_source=chatgpt.com, accessed on 23 February 2026) interface was used to select target test locations within the sample display area, with the software automatically positioning each selected point under the microscope. Fine focus adjustment was subsequently performed in the microscope video display window to achieve a clear and sharp image of the sample surface, ensuring precise indentation placement.

**Figure 5:**
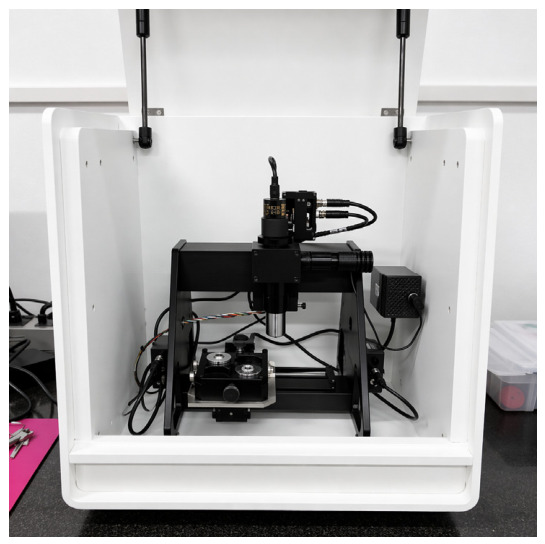
Nanoindentation test.

For each asphalt binder, the elastic modulus was estimated from the average value of at least four indentation test points and was used for comparative analysis of local micromechanical response among the binder systems. Given the limited number of indentation points, these results are interpreted as supplementary comparative evidence rather than a standalone statistical characterization. The calculation was performed using the following equation:

(1) $$E=\frac{1}{n}\overset{n}{\underset{i=1}{\sum }}x_{i}$$

where E is the average elastic modulus calculated from the four indentation points at each test frequency, and $x_{i}$ represents the storage modulus measured at each individual point. The average elastic modulus values of the asphalt binders at different frequencies were subsequently determined. Representative test results are presented in Figure 5.

#### 2.2.7. Road Performance Test

**Figure 6:**
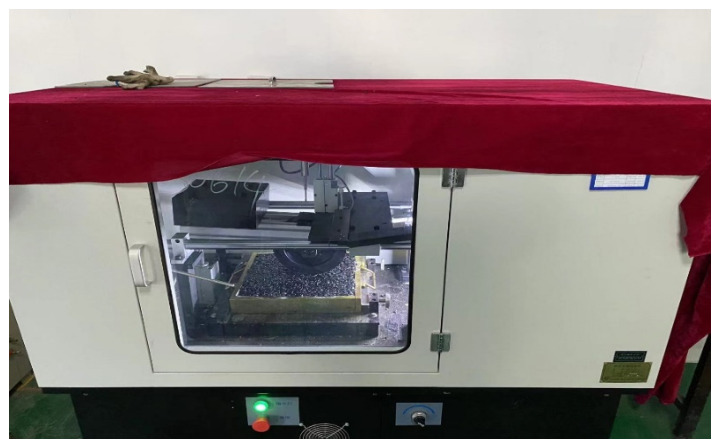
Rutting test.

The high-temperature rutting resistance, low-temperature cracking resistance, and moisture stability of AC-13 warm-mix asphalt mixtures containing basalt fiber or basalt fiber powder were evaluated through rutting tests at 60 °C, three-point bending tests at −10 °C, and immersion Marshall tests, respectively. These tests were conducted to investigate the effects of basalt fiber and basalt fiber powder addition on the performance of warm-mix asphalt mixtures. All mixture performance tests were performed in triplicate, and the reported values represent the average of three measurements; therefore, the results should be interpreted as comparative trends under the present test conditions. Because the mixture tests were conducted with only three replicates per group, the reported results should be interpreted as preliminary comparative evidence, and more extensive statistical validation is needed in future work. The rutting test setup is illustrated in Figure 6.

## 3. Results and Discussion

### 3.1. High-Temperature Rheological Properties

**Figure 7:**
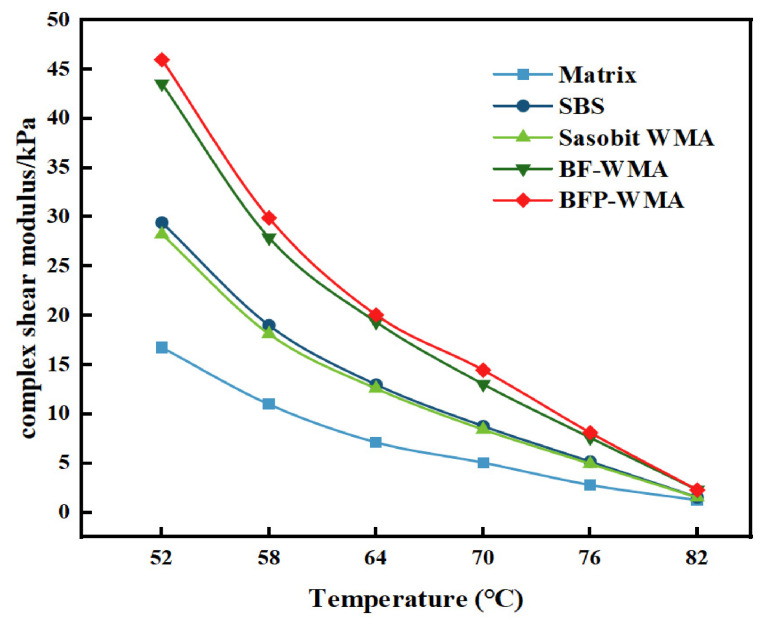
Complex shear modulus–temperature change graph.

**Figure 8:**
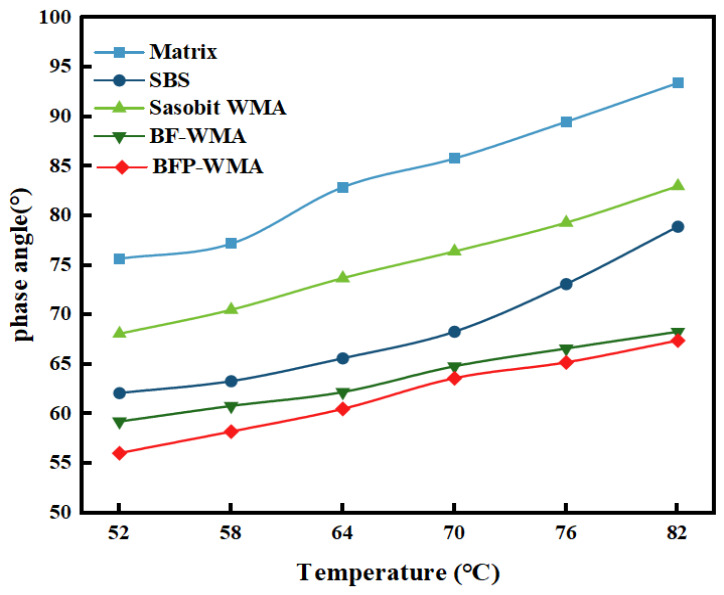
Phase angle-temperature change graph.

**Figure 9:**
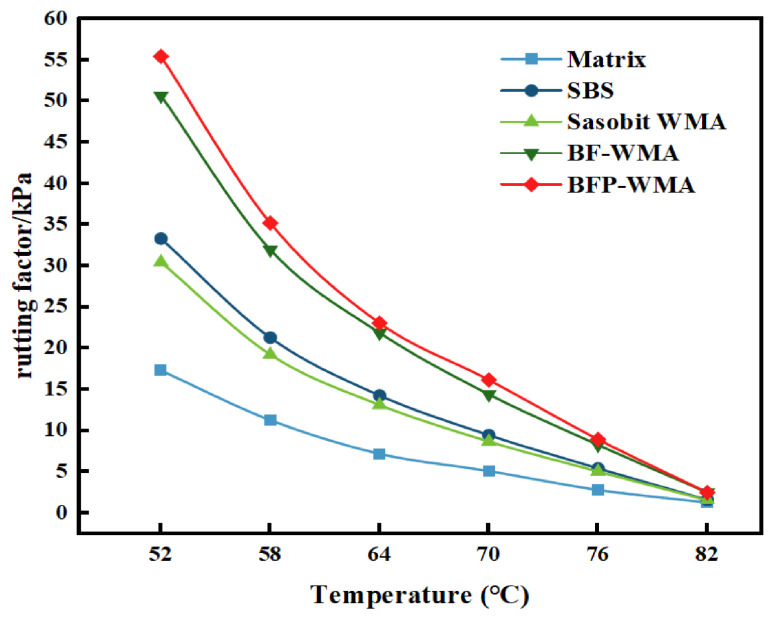
Rutting factor–temperature relationship.

The temperature-dependent rheological properties of the five asphalt binders—base asphalt, SBS-modified asphalt, Sasobit warm-mix asphalt (SWMA), basalt fiber-modified WMA (BF-WMA), and basalt fiber powder-modified WMA (BFP-WMA)—were evaluated via DSR temperature sweep tests. The complex shear modulus (G*), phase angle (*δ*), and rutting factor (G*/sin *δ*) as functions of temperature are presented in Figure 7 and Figure 8, and Figure 9, respectively.

The high-temperature rheological properties of the five asphalt binders—base asphalt, SBS-modified asphalt, Sasobit warm-mix asphalt (SWMA), basalt fiber-modified WMA (BF-WMA), and basalt fiber powder-modified WMA (BFP-WMA)—were evaluated via temperature sweep tests from 52 °C to 82 °C. As shown in Figure 7, Figure 8 and Figure 9, the complex shear modulus (G*), phase angle (*δ*), and rutting factor (G*/sin *δ*) exhibited temperature-dependent trends that reflect the viscoelastic behavior of each binder.

In the low-temperature region (52 °C~64 °C), all binders exhibited relatively high G* and low *δ* values, indicating a predominantly elastic response and strong resistance to deformation. As the temperature increased to the high-temperature region (70 °C~82 °C), G* decreased markedly while δ increased, reflecting a transition to viscous-dominated behavior and a corresponding reduction in deformation resistance.

The incorporation of modifiers improved the high-temperature performance of the binders under the present test conditions. At 58 °C, SWMA exhibited a 65% increase in G* relative to the base asphalt and was only 5% lower than SBS-modified asphalt, while at 76 °C, BFP-WMA showed the highest rutting factor, exceeding BF-WMA by 8.1% and outperforming the SBS-modified binder. This suggests that while Sasobit reduces binder viscosity at mixing temperatures, its wax crystallization at service temperatures contributes to increased stiffness. Notably, BFP-WMA exhibited G* values exceeding those of SBS-modified asphalt, which may be attributed to the more uniform dispersion of rigid micronized particles and the resulting improvement in interfacial interactions within the binder. The improved stiffness is likely due to the combined effects of these rigid microparticles and enhanced interfacial interactions, which restrict molecular mobility and delay high-temperature softening.

The phase angle data further elucidate the viscoelastic response. The δ values ranked in the following order: base asphalt > SWMA > SBS-modified asphalt > BF-WMA > BFP-WMA. The lowest δ observed for BFP-WMA suggests that the finer and more uniformly dispersed BFP may improve interfacial organization within the binder and restrict molecular mobility, thereby reducing viscous deformation and contributing to enhanced elastic recovery under shear loading.

The rutting factor (G*/sin *δ*) provides a quantitative measure of high-temperature deformation resistance. At the critical temperature of 76 °C, BFP-WMA exhibited the highest rutting factor among the tested binders. Its value was 8.1% higher than that of BF-WMA and also higher than that of the SBS-modified binder, indicating a favorable high-temperature deformation resistance trend under the tested conditions. In contrast, SWMA showed a moderate reduction in rutting factor at 76 °C, which may be attributed to a gradual decrease in wax crystallization efficiency or partial phase transition of the wax at elevated temperatures, leading to diminished reinforcement.

In summary, basalt fiber powder improves the high-temperature stiffness and elastic recovery of asphalt under the tested conditions, likely owing to its rigid geometry and improved dispersion/interfacial interaction. Meanwhile, the high-temperature performance of Sasobit-based systems appears to be influenced by the thermal stability and phase behavior of the wax component at elevated temperatures.

### 3.2. Low-Temperature Rheological Properties

**Figure 10:**
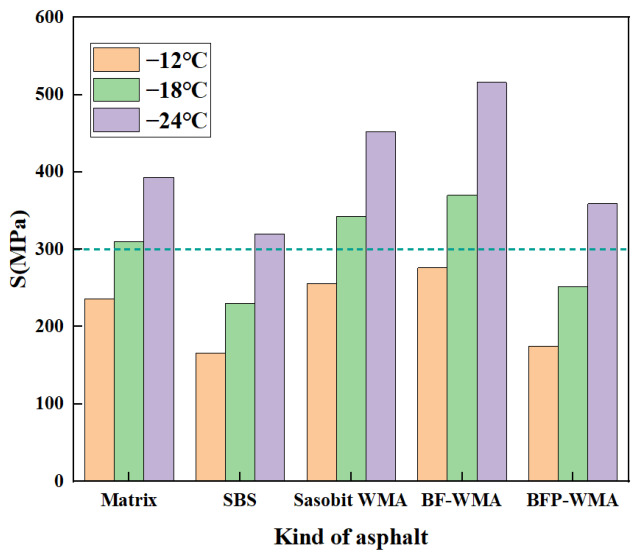
Asphalt creep modulus–temperature curve.

**Figure 11:**
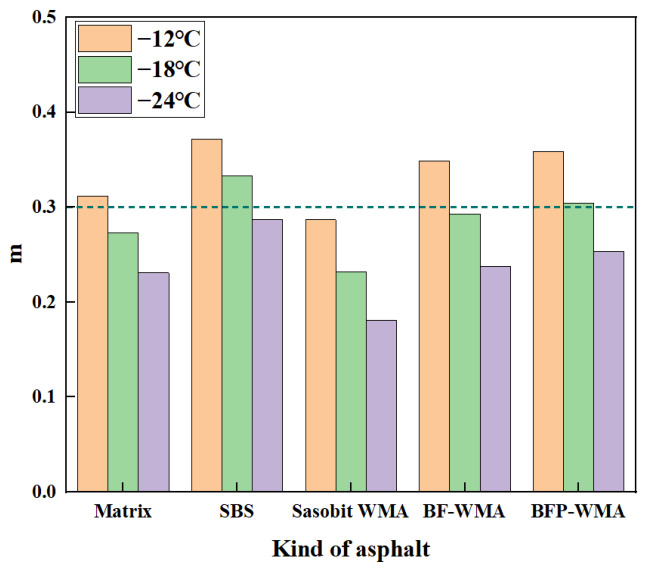
Asphalt creep rate–temperature curve.

The low-temperature performance of the asphalt binders was evaluated using the criteria of creep stiffness *S* ≤ 300.0 MPa and creep rate *m* ≥ 0.3, as specified in relevant standards [31,32,33]. The temperature-dependent *S* and *m*-value curves for the five binders are presented in Figure 10 and Figure 11, respectively.

As shown in Figure 10, decreasing the test temperature from −12 °C to −24 °C resulted in a marked increase in creep stiffness (*S*) for all asphalt binders, indicating enhanced brittleness and deteriorated low-temperature deformation resistance. At all three test temperatures, Sasobit warm-mix asphalt (SWMA) and basalt fiber-modified WMA (BF-WMA) exhibited higher stiffness than base asphalt, reflecting inferior low-temperature deformation resistance. Among these, BF-WMA showed the highest S values and the poorest deformation resistance. Specifically, at −12 °C, the *S* value of base asphalt (236.2 MPa) was 8.2% and 14.5% lower than those of SWMA and BF-WMA, respectively. At −24 °C, the *S* value of base asphalt (393.5 MPa) was 13.1% and 24.0% lower than those of SWMA and BF-WMA, respectively. These results indicate that as temperature decreases, the brittleness of warm-mix modified asphalts—particularly those modified with conventional basalt fibers—becomes more pronounced, widening the performance gap relative to base asphalt.

Notably, basalt fiber powder-modified WMA (BFP-WMA) exhibited a distinctly different trend. Its creep stiffness was consistently lower than that of the base asphalt across all three test temperatures, with reductions of 25.8%, 18.7%, and 8.7% at −12 °C, −18 °C, and −24 °C, respectively. These results indicate that the incorporation of micronized basalt fiber powder improves the low-temperature deformation resistance of warm-mix asphalt and may partially offset the stiffening effect associated with Sasobit and conventional basalt fiber modification.

As shown in Figure 11, the creep rate (*m*-value) of all asphalt binders decreased progressively as the test temperature dropped from −12 °C to −24 °C, indicating a general reduction in viscoelasticity and stress relaxation capacity. Across the entire temperature range, the *m*-value of Sasobit warm-mix asphalt (SWMA) remained consistently lower than that of base asphalt, reflecting its diminished stress relaxation ability at low temperatures.

The incorporation of basalt fibers improved the *m*-value of warm-mix asphalt relative to base asphalt by 11.8%, 7.0%, and 3.0% at −12 °C, −18 °C, and −24 °C, respectively. This demonstrates that fiber addition enhances low-temperature stress relaxation. However, the improvement diminished progressively with decreasing temperature. This trend may be attributed to progressive stiffening of the asphalt matrix at lower temperatures, which restricts effective stress redistribution and limits the toughening contribution of the fibers. Under such conditions, the brittle matrix fails to transmit stress efficiently, thereby weakening the fiber toughening effect (as evidenced by the reduction in improvement rate from 11.8% to 3.0%).

Notably, the replacement of conventional basalt fibers with basalt fiber powder (BFP) resulted in a more pronounced enhancement of the *m*-value, with increases of 15.1%, 11.3%, and 9.5% at the respective temperatures. This indicates that BFP is superior to conventional fibers in improving low-temperature stress relaxation capacity, and its performance degradation with decreasing temperature is slower. This advantage is primarily attributed to the higher specific surface area and superior dispersibility of BFP, which enhance interfacial interaction with the asphalt matrix, facilitate low-temperature stress transfer, and improve the material’s ability to inhibit microcrack propagation.

In summary, the BBR test results reveal distinct low-temperature behavior among the modified binders. Conventional basalt fiber-reinforced warm-mix asphalt (BF-WMA) exhibits a trade-off between stiffness and stress relaxation: increased creep stiffness (indicating reduced deformation resistance) alongside improved *m*-value (reflecting enhanced stress relaxation). In contrast, the incorporation of basalt fiber powder (BFP) simultaneously improves both deformation resistance and stress relaxation capacity, as evidenced by reduced stiffness modulus and increased *m*-value. This synergistic enhancement is attributed to the high specific surface area and superior dispersibility of BFP, which optimize interfacial stress transfer efficiency and microcrack inhibition within the asphalt matrix.

Additionally, at −12 °C, the performance of BFP-WMA closely approaches that of SBS-modified asphalt, with a stiffness modulus difference of only +5.4% and an m-value difference of −3.6%. This indicates that BFP-WMA can achieve SBS-comparable low-temperature performance at moderately low temperatures. However, this similarity is temperature-dependent and becomes less pronounced at lower temperatures. As the temperature drops to −24 °C, the performance gap widens significantly, with the stiffness modulus difference expanding to +12.1% and the m-value difference to −11.8%, indicating that BFP-WMA still remains inferior to SBS-modified asphalt under more severe low-temperature conditions.

It should be noted that the improved low-temperature relaxation observed in BBR tests does not contradict the higher elastic modulus obtained from nanoindentation measurements. These two characterization methods probe different scales and loading regimes: nanoindentation captures localized, high-frequency micromechanical response, whereas BBR reflects global, low-frequency flexural relaxation behavior. The apparent discrepancy thus arises from the multiscale nature of the material’s viscoelastic response, underscoring the importance of complementary characterization techniques in understanding the performance of modified asphalt binders.

### 3.3. Asphalt Dispersion Uniformity

**Figure 12:**
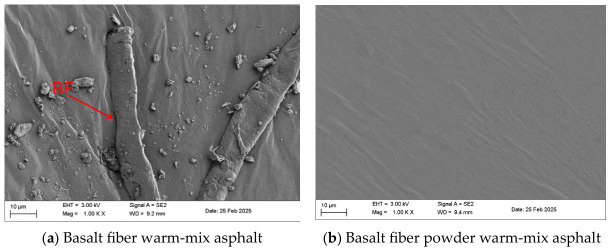
Micrographic morphology of basalt fiber and fiber powder warm-mix asphalt.

Scanning electron microscopy (SEM) was employed to examine the microstructure and dispersion characteristics of basalt fiber and basalt fiber powder within the warm-mix asphalt binder. The SEM observations provide insight into the distribution of the modifiers and their interfacial interaction with the asphalt matrix [34]. Figure 12 presents SEM micrographs revealing distinct microstructural differences between basalt fiber-modified warm-mix asphalt (BF-WMA) and basalt fiber powder-modified warm-mix asphalt (BFP-WMA). In BF-WMA, undispersed fiber bundles are clearly visible, indicating non-uniform dispersion of conventional fibers within the asphalt matrix. In contrast, BFP-WMA exhibits a homogeneous distribution of micron-sized particles, with no observable phase separation. This suggests that micronization through ball milling enhances dispersion uniformity and may improve interfacial compatibility between the fiber powder and asphalt.

The improved dispersion of BFP in BFP-WMA may be related to two factors. First, mechanical grinding increases the specific surface area of the fibers, which may facilitate a more effective physical interaction with asphalt during high-speed shear mixing. Second, high-temperature mixing may promote asphalt wetting and interfacial contact with the fiber powder surface, thereby contributing to improved microstructural continuity.

It is worth noting that localized micron-scale wrinkles (approximately 0.8–1.5 μm in depth) and interface gaps (width < 300 nm) are still observable in BFP-WMA. These features may reflect local heterogeneity in the binder–modifier system and indicate that the interface is not completely uniform at the microscale. Nevertheless, BFP-WMA exhibits a more homogeneous reinforcing morphology overall, which may contribute to improved structural continuity. This more uniform dispersion state may enhance the material’s high-temperature deformation resistance and low-temperature crack resistance by restricting the plastic flow of asphalt molecular chains and promoting more effective stress redistribution.

### 3.4. Nanoscale Mechanical Properties

**Figure 13:**
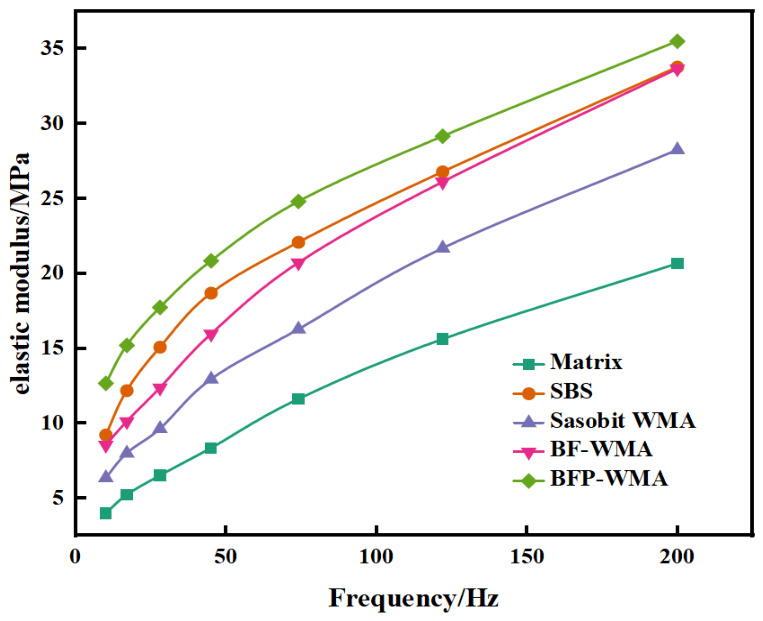
Asphalt elastic modulus time–frequency elastic modulus curve.

The elastic modulus of the asphalt binders, a key indicator of their elasticity and deformation resistance, was characterized using nanoindentation (NI). Figure 13 shows the frequency-dependent elastic modulus of the five binder systems. The figure reveals distinct differences in the frequency-dependent elastic modulus among the five asphalt binders. Sasobit warm-mix asphalt (SWMA) exhibited a significantly lower elastic modulus compared to SBS-modified asphalt and the fiber-modified groups, with the disparity becoming more pronounced at higher frequencies. This indicates that warm-mix additive alone is insufficient to effectively enhance the stiffness of the asphalt binder. Notably, in the high-frequency range, basalt fiber powder-modified WMA (BFP-WMA) displayed a higher elastic modulus than basalt fiber-modified WMA (BF-WMA). This superior performance is attributed to the uniform dispersion and high specific surface area of BFP, which enhance fiber–asphalt interfacial bonding and matrix continuity, thereby providing greater resistance to high-frequency dynamic loading.

The nanoindentation results corroborate the findings from DSR testing, reinforcing the conclusion that basalt-based modifiers enhance the stiffness of asphalt binders. At 25 °C and 10 Hz, the shear modulus values derived from nanoindentation ($G_{\mathrm{NI}}$) and DSR ($G_{\mathrm{DSR}}$) exhibited consistent trends across the five binder systems (Table 5). Sasobit addition reduced both $G_{\mathrm{NI}}$ and $G_{\mathrm{DSR}}$, while the inclusion of basalt fibers and basalt fiber powder increased both parameters. These observations confirm that the stiffening effect of basalt modifiers extends from the macroscale to the microscale, reflecting enhanced local micromechanical stiffness under high-frequency loading.

Although $G_{\mathrm{DSR}}$ values were consistently 2.2 to 2.3 times higher than $G_{\mathrm{NI}}$ values, this quantitative difference is expected given the distinct testing regimes: DSR measures bulk viscoelastic response under shear deformation at relatively large strain amplitudes, whereas nanoindentation probes localized elastic response under compressive loading at nanoscale contact areas. The conversion between E and G (Equation (2)) provides a theoretical basis for comparing these measurements, but inherent differences in scale and deformation mode preclude perfect quantitative agreement. Nevertheless, the two techniques showed a consistent relative ranking among the tested binder systems, supporting the use of nanoindentation as a complementary tool for interpreting the micromechanical origin of macroscale rheological behavior.

(2) E=2G(1+ν)

where *G* is the shear modulus of asphalt, *E* is the elastic modulus obtained from nanoindentation, and *ν* is Poisson’s ratio of asphalt.

### 3.5. Research on the Road Performance of Warm-Mix Asphalt Mixtures

**Figure 14:**
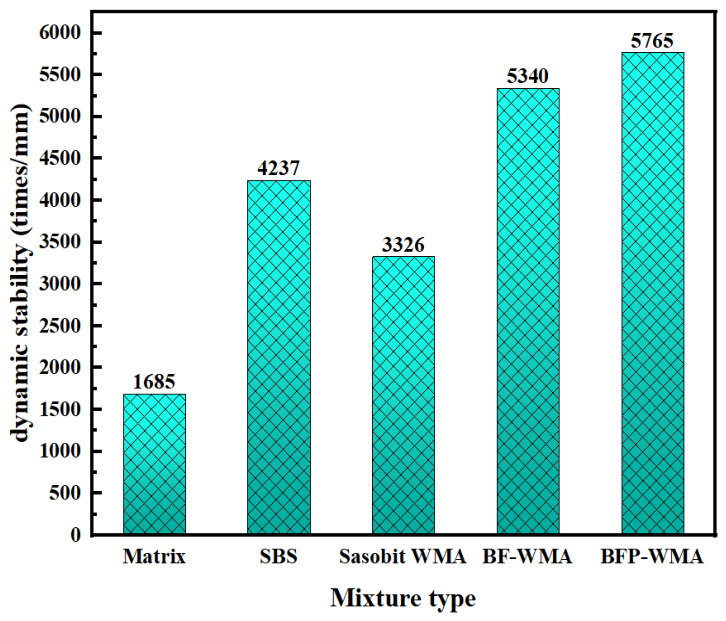
Results of wheel track test.

**Figure 15:**
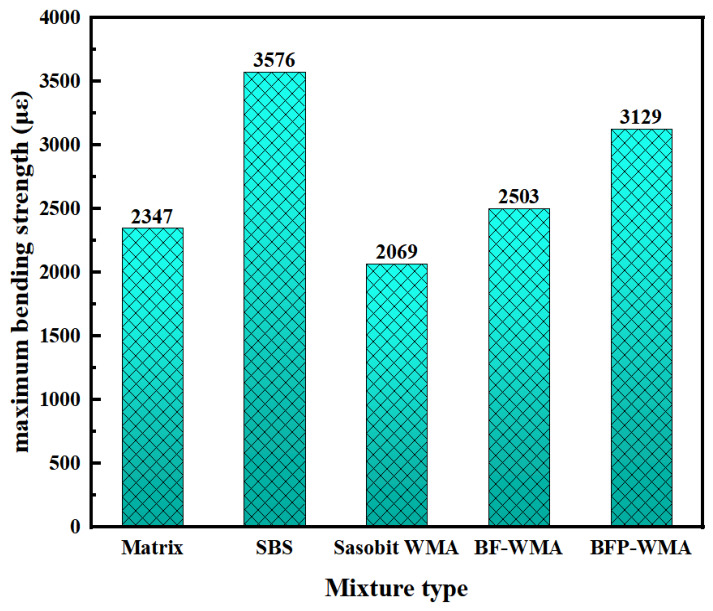
Low-temperature crack resistance test results.

**Figure 16:**
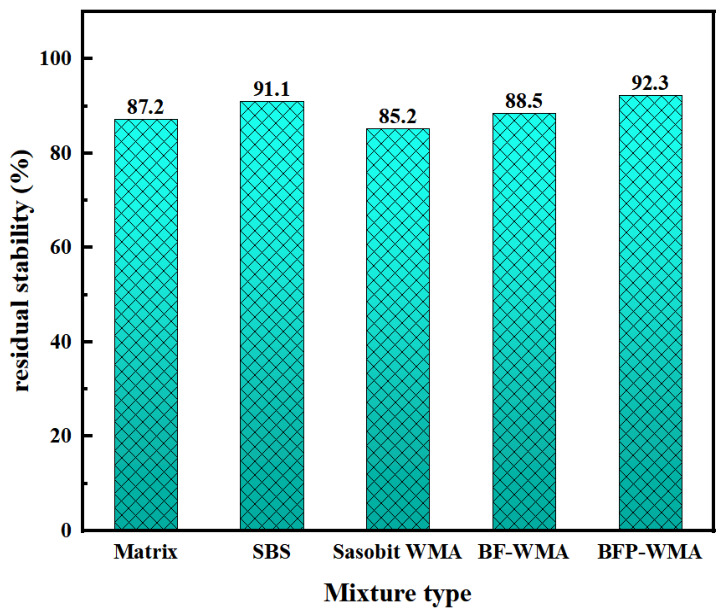
Results of the Marshall test after immersion in water.

The optimum asphalt contents for the AC-13 mixtures prepared with base asphalt, SBS-modified asphalt, Sasobit warm-mix asphalt (SWMA), basalt fiber-modified WMA (BF-WMA), and basalt fiber powder-modified WMA (BFP-WMA) were determined to be 4.2%, 4.5%, 4.1%, 4.7%, and 4.9%, respectively. The effects of different modification mechanisms on mixture performance were evaluated through high-temperature rutting tests at 60 °C, low-temperature three-point bending tests at −10 °C, and immersion Marshall tests for moisture susceptibility. The test results are presented in Figure 14 and Figure 15, and Figure 16, respectively.

The dynamic stability (DS) test results are presented in Figure 14. The five asphalt mixtures showed different levels of DS improvement relative to the base asphalt mixture, indicating differences in the effect of each modifier under the present test conditions. Specifically, the Sasobit warm-mix additive increased DS by 97.4% compared with the base asphalt mixture, which was lower than the increases observed for the SBS-modified (151.5%), basalt fiber-modified (217.0%), and basalt fiber powder-modified (242.2%) warm-mix systems.

This disparity stems from the fundamentally different mechanisms by which these modifiers enhance high-temperature performance. Sasobit primarily improves workability and provides limited deformation resistance through physical viscosity reduction and partial hardening but does not fundamentally alter the high-temperature viscoelastic properties of the asphalt binder. In contrast, SBS forms a three-dimensional polymer network that significantly enhances elastic recovery and resistance to permanent deformation. Notably, the basalt fiber-modified systems represent a composite modification strategy that synergistically combines the workability benefits of Sasobit with the macro-reinforcement and bridging effects of basalt fibers. The fiber network effectively compensates for the inherent limitations of Sasobit in high-temperature deformation resistance and constrains aggregate slippage under load. This synergistic effect is most pronounced in the basalt fiber powder-modified system, which achieved the highest DS increase (242.2%), highlighting the potential of composite modification strategies for enhancing rutting resistance in warm-mix asphalt pavements. These findings provide mechanistic insight into morphology-regulated reinforcement strategies for optimizing high-temperature performance.

The maximum flexural strain test results, presented in Figure 15, reveal the influence of different modifiers on the low-temperature cracking resistance of the AC-13 mixtures. Compared to base asphalt, the addition of Sasobit reduced the maximum flexural strain by 11.8%, confirming that the warm-mix additive compromises low-temperature performance. When conventional basalt fibers were incorporated into the Sasobit system, the flexural strain increased by 6.6% relative to base asphalt, indicating that fibers can partially offset the deterioration induced by Sasobit. More notably, the incorporation of basalt fiber powder resulted in a 33.3% increase in flexural strain, indicating an improved low-temperature bending deformation capacity under the tested conditions. This improvement is attributed to the high specific surface area and excellent dispersibility of BFP, which promote more effective interfacial interaction and toughening within the asphalt matrix.

Despite this significant enhancement, the flexural strain of the BFP-modified warm-mix system remained 12.5% lower than that of SBS-modified asphalt. This indicates that while BFP provides substantial physical toughening, its ability to improve low-temperature cracking resistance does not yet match that of polymer modification. Therefore, in pavement design for severely cold regions, the high stiffness of BFP-modified warm-mix asphalt may offer advantages in resisting thermal stress, but its flexural strain capacity should be carefully evaluated to mitigate the risk of brittle failure under extreme low-temperature conditions.

The residual Marshall stability test results, presented in Figure 16, demonstrate that basalt fiber powder-modified warm-mix asphalt (BFP-WMA) exhibits superior moisture resistance among all mixtures, with a residual stability of 92.3%. This value is 4.3%, 5.8%, 8.3%, and 1.3% higher than those of conventional basalt fiber-modified WMA (BF-WMA), base asphalt, Sasobit warm-mix asphalt (SWMA), and SBS-modified asphalt mixtures, respectively.

This superior moisture stability may be related to the multiscale effects of micronized basalt fiber powder. First, the micron-sized particles may help reduce interstitial voids within the mixture, which could be beneficial for limiting moisture ingress. Second, the higher specific surface area and improved dispersion of BFP may promote more effective contact within the asphalt–aggregate system. Third, the fine modifier morphology may contribute to improved interfacial stability under water exposure. In addition, the viscosity-reducing effect of the Sasobit warm-mix process may facilitate more uniform dispersion of the fiber powder within the mixture. Collectively, these factors may contribute to the higher moisture resistance observed for BFP-WMA under the present test conditions.

In summary, the warm-mix basalt fiber powder composite system (BFP-WMA) exhibits the most balanced overall performance among the tested mixtures, achieving the highest high-temperature rutting resistance (DS) and superior moisture stability (residual stability). While its low-temperature cracking resistance is significantly improved by the incorporation of BFP, it remains slightly inferior to that of SBS-modified asphalt. Nonetheless, this system shows promising potential for improving the overall performance of warm-mix asphalt through fiber micronization under the present experimental conditions.

## 4. Conclusions

(1) Basalt fiber powder (BFP) was successfully prepared via ball milling and uniformly dispersed in Sasobit-based warm-mix asphalt. SEM observations confirmed that the micronization process effectively mitigated fiber agglomeration and promoted a homogeneous distribution within the asphalt matrix, contributing to a denser and more stable internal microstructure.

(2) BFP improved the high-temperature rutting resistance of Sasobit-modified WMA, with performance approaching that of SBS-modified asphalt across the tested temperature range.

(3) The incorporation of BFP improved the low-temperature deformation capacity of warm-mix asphalt and partially offset the stiffness increase induced by Sasobit. However, this beneficial effect diminished at extremely low temperatures.

(4) At the mixture level, BFP-modified warm-mix asphalt exhibited superior rutting resistance and moisture stability, reflecting improved aggregate–binder interfacial bonding and enhanced structural integrity. Although its low-temperature cracking resistance remained slightly lower than that of SBS-modified mixtures, it demonstrated a marked improvement over conventional warm-mix systems.

(5) The proposed BFP modification strategy is compatible with conventional mixing procedures and offers a practical pathway for enhancing the performance of warm-mix asphalt. However, its practical application may still be constrained by the additional cost, energy demand, and scale-up challenges associated with BFP preparation. Further techno-economic evaluation and field validation are needed before large-scale implementation.

## Figures and Tables

**Table 1:** Technical specifications for matrix asphalt.

Testing Item		Unit	Test Result	Technical Index
Needle penetration (100 g, 5 s, 25 °C)		0.1 mm	73.2	60–80
Softening point		°C	49.7	≥46
Ductility (10 °C 5 cm/min)		cm	73.0	≥25
Penetration index			−1.1	−1.5~1.0
Dynamic viscosity		Pa·s	271	≥180
Flash point		°C	280	≥260
Wax content		%	0.8	≤2.2
Density (15 °C)		g/cm^3^	1.054	Actual measurement
After TFOT (163 °C, 5 h)	Mass variation	%	−0.255	≤±0.8
	Residual penetration ratio (25 °C)	%	68.3	≥61
	Residual ductility (10 °C)	cm	10.6	≥6

**Table 2:** SBS-modified asphalt technical specifications.

Testing Item		Unit	Test Result	Specification Requirement
Needle penetration (100 g, 5 s, 25 °C)		0.1 mm	53	≥50
Softening point		°C	76.0	≥75
Ductility (5 °C 5 cm/min)		cm	25	≥20
Elastic recovery (25 °C)		%	92	≥90
Brookfield rotational viscosity (135 °C)		Pa·s	2.355	2.2~3.0
Density (25 °C)		g/cm^3^	1.004	Actual measurement
After TFOT (163 °C, 5 h)	Mass variation	%	−0.018	±1.0
	Residual penetration ratio (25 °C)	%	68.2	≥65
	Residual ductility (10 °C)	cm	17	≥15

**Table 3:** Technical specifications of Sasobit warm mix additive.

Project	Appearance	Water Solubility	pH Value	Density/g·cm^−3^	Freezing Poin/°C	Flash Point/°C
Typical value	Milky white solid	Insoluble	Neutral	0.94	100	290

**Table 4:** Basalt fiber parameters.

Parameters	Basalt Fiber
Diameter (μm)	9
Common length (mm)	6
Specific gravity (g/cm^3^)	2.56
Tensile strength (MPa)	3000
Elastic modulus (GPa)	89
Elongation at break (%)	3.1

**Table 5:** Asphalt NI and DSR shear modulus.

Asphalt Type	$G_{\mathrm{NI}}$/MPa	$G_{\mathrm{DSR}}$/MPa
Matrix	1.22	2.76
SBS	1.62	3.69
Sasobit WMA	1.33	3.02
BF-WMA	2.21	5.17
BFP-WMA	3.34	7.66

## Data Availability

The original contributions presented in this study are included in the article. Further inquiries can be directed to the corresponding authors.

## References

[1] Rubio M.C., Martínez G., Baena L., Moreno F. (2012). Warm mix asphalt: An overview. J. Clean. Prod..

[2] Almeida-Costa A., Benta A. (2016). Economic and environmental impact study of warm mix asphalt compared to hot mix asphalt. J. Clean. Prod..

[3] Jamshidi A., Hamzah M.O., You Z. (2013). Performance of warm mix asphalt containing Sasobit^®^: State-of-the-art. Constr. Build. Mater..

[4] Behnood A., Karimi M.M., Cheraghian G. (2020). Coupled effects of warm mix asphalt (WMA) additives and rheological modifiers on the properties of asphalt binders. Clean. Eng. Technol..

[5] Gao J., Yan K., He W., Yang S., You L. (2018). High temperature performance of asphalt modified with Sasobit and Deurex. Constr. Build. Mater..

[6] Rossi D., Filippi S., Merusi F., Giuliani F., Polacco G. (2013). Internal structure of bitumen/polymer/wax ternary mixtures for warm mix asphalts. J. Appl. Polym. Sci..

[7] Yuan H., Liu J., Ding H., Xie Q., Qiu Y. (2024). Evaluation of physical hardening of wax-based warm mix asphalt binders from low-temperature rheological properties. Constr. Build. Mater..

[8] Yan K.Z., Xu H.B., Zhang H.L. (2013). Effect of mineral filler on properties of warm asphalt mastic containing Sasobit. Constr. Build. Mater..

[9] Guo Y., Tataranni P., Sangiorgi C. (2023). The use of fibres in asphalt mixtures: A state of the art review. Constr. Build. Mater..

[10] Morea F., Zerbino R. (2020). Incorporation of synthetic macrofibres in Warm Mix Asphalt. Road Mater. Pavement Des..

[11] Adepu R., Ramayya V.V., Mamatha A., Ram V.V. (2023). Fracture studies on basalt fiber reinforced asphalt mixtures with reclaimed asphalt pavement derived aggregates and warm mix additives. Constr. Build. Mater..

[12] Hui Y., Men G., Xiao P., Tang Q., Han F., Kang A., Wu Z. (2022). Recent advances in basalt fiber reinforced asphalt mixture for pavement applications. Materials.

[13] Zhao H., Guan B., Xiong R., Zhang A. (2020). Investigation of the performance of basalt fiber reinforced asphalt mixture. Appl. Sci..

[14] Zhou H., Li M., Jin G., Guo M., Jiang Y. (2025). Combined effects of basalt fiber geometrical characteristics on pavement performance of asphalt mixtures. PLoS ONE.

[15] Lou K., Wu X., Xiao P., Kang A., Wu Z., Xia Y. (2021). Comprehensive study about effect of basalt fiber, gradation, nominal maximum aggregate size and asphalt on the anti-cracking ability of asphalt mixtures. Appl. Sci..

[16] Tanzadeh R., Tanzadeh J., Honarmand M., Tahami S.A. (2019). Experimental study on the effect of basalt and glass fibers on behavior of open-graded friction course asphalt modified with nano-silica. Constr. Build. Mater..

[17] Cheng P., Yi J., Guo S., Pei Z., Feng D. (2022). Influence of fiber dispersion and distribution on flexural tensile properties of asphalt mixture Based on finite element simulation. Constr. Build. Mater..

[18] Cheng X., Liu J., Han C., Zhang X., Wu Z. (2023). Silane coupling agent impact on surface features of modification of basalt fibers and the rheological properties of basalt fiber reinforced asphalt. Constr. Build. Mater..

[19] Xiang Y., Xie Y., Long G. (2018). Effect of basalt fiber surface silane coupling agent coating on fiber-reinforced asphalt: From macro-mechanical performance to micro-interfacial mechanism. Constr. Build. Mater..

[20] Ren D., Luo W., Su S., Wang Z., Kong L., Ai C. (2024). Study on crack resistance of basalt fiber reinforced asphalt mixture modified by titanate coupling agent based on digital image correlation. Constr. Build. Mater..

[21] Zhu M., Zhu M., Zhai R., Zhu W., He J. (2025). Research Progress on the Surface Modification of Basalt Fibers and Composites: A Review. Materials.

[22] Cai C., Lou K., Qian F., Xiao P. (2024). Influence of Basalt Fiber Morphology on the Properties of Asphalt Binders and Mixtures. Materials.

[23] Kou C., Chen Z., Kang A., Zhang M., Wang R. (2022). Rheological behaviors of asphalt binders reinforced by various fibers. Constr. Build. Mater..

[24] Wang L., Liu Z., Li C., Tian Z., Hu X. (2025). Cracking characteristics of warm-mix recycled fiber asphalt mixture under Mode I, Mode III, and mixed-Mode I/III based on acoustic emission technology. Constr. Build. Mater..

[25] Li C., Zhang Y., Wang L., Tian Z., Zhao M., Li Z. (2026). Effects of basalt fiber morphology on the crack resistance and damage behavior of warm-mix recycled asphalt mixtures. Constr. Build. Mater..

[26] Gui W., Hu X., Wang L., Li C., Zhan Y., Zhang F., Tian Z. (2025). Investigating fracture behaviors of fiber-reinforced warm-mixed recycled SBS modified asphalt mixtures using the DIC and AE techniques under different loading modes. Theor. Appl. Fract. Mech..

[27] (2011). Standard Test Methods of Bitumen and Bituminous Mixtures for Highway Engineering.

[28] Pang J., Chen Y., Jing L., Song H., Liu Z. (2025). Performance Evaluation of Warm-Mix Asphalt Binders with an Emphasis on Rutting and Intermediate-Temperature Cracking Resistance. Materials.

[29] Bilema M., Wah Yuen C., Alharthai M., Hazim Al-Saffar Z., Oleiwi Aletba S.R., Md Yusoff N.I. (2023). Influence of warm mix asphalt additives on the physical characteristics of crumb rubber asphalt binders. Appl. Sci..

[30] Shi X., Si C., Yan K., Zhu Y. (2024). Research on the low-temperature performance of basalt fiber-rubber powder modified asphalt mixtures under freeze-thaw in large temperature differences region. Sci. Rep..

[31] Al-Khateeb G.G., Sukkari A., Ezzat H., Nasr E., Zeiada W. (2024). Rheology of crumb rubber-modified warm mix asphalt (WMA). Polymers.

[32] Saed S.A., Ziari H., Kamboozia N., Dehaghi E.A. (2025). Rheological and aging performance of reclaimed asphalt binders modified by warm mix additive and recycling agent. Sci. Rep..

[33] Kim Y.S., Büchner J., Wistuba M.P., Agudo J.R., Rochlani M., Schäffler M. (2022). Asphalt binder testing at low temperature: Three-point bending beam test in dynamic shear rheometer. Front. Mater..

[34] Mazumder M., Ahmed R., Ali A.W., Lee S.J. (2018). SEM and ESEM techniques used for analysis of asphalt binder and mixture: A state of the art review. Constr. Build. Mater..

